# Correction: Rescaling of Spatio-Temporal Sensing in Eukaryotic Chemotaxis

**DOI:** 10.1371/journal.pone.0171765

**Published:** 2017-02-06

**Authors:** The PLOS ONE Staff

In the author contributions, Yohei Kondo should be listed in the Software section instead of Keita Kamino. The publisher apologizes for this error.
