# Supplementary material for: Rescaling of Spatio-Temporal Sensing in Eukaryotic Chemotaxis
Source: PLoS One. 2016 Oct 28;11(10):e0164674. doi: 10.1371/journal.pone.0164674 (PMC5085096; doi:10.1371/journal.pone.0164674)
Supplement: S1 Text — (PDF) [file pone.0164674.s001.pdf]

Supporting Text S1

Rescaling of spatio-temporal sensing in eukaryotic chemotaxis

Keita Kamino, [1¶*] Yohei Kondo, [2¶*]

[1]FOM Institute AMOLF, Amsterdam, Netherlands, [2]Graduated school of Informatics, Kyoto University, Kyoto, Japan,

*Corresponding author

¶ These authors contributed equally to this work.

Earlier models for the eukaryotic gradient sensing system and their symmetric properties

In the main text, we have shown that, based on the LEGI-framework [1], spatial and temporal FCDs can be achieved respectively by two different scale-invariant properties in the governing equations, i.e., the spatial and temporal FCD symmetry (Fig. 5a). The two symmetries can be implemented in a mathematical model independently of each other (Fig. 5). The scale-invariant LEGI model (Eqs. 1) satisfies the two symmetries at the same time, and thus the model shows characteristic rescaled responses to stimuli that change in both space and time like traveling wave stimuli (Fig. 6). Among the two symmetric properties, the temporal FCD symmetry and the system-level consequence, i.e., temporal FCD, have been investigated extensively based on ordinary differential equation systems [2,3] and applied to directional sensing in spatially-homogeneous stimuli [4]. Concerning spatial FCD, although several LEGI-based models have the spatial FCD property as pointed out by Nakajima et al [5], the symmetric properties of the models, to our best knowledge, have not been studied explicitly. Here, we analyze representative LEGI-based models in the literature in terms of their symmetric properties.

(i) Levchenko-Iglesias models [6]

In the pioneering work by Levchenko and Iglesias [6], LEGI-based models have been proposed to describe the response properties of the gradient sensing system. They have assumed that the signaling component characterizing the response is found in both an active, $R*$, and an inactive, R, states. The conversion from $R*$ to R is mediated by the inactivator I and the opposite reaction is mediated by the activator A. Both of the activator A and inactivator I are activated by chemoattractant S. The first model equations they have proposed (Fig. 3A in [6]) are

$$\frac{\partial A}{\partial t} = -k_{-A}A + k_A S,$$

$$\frac{\partial I}{\partial t} = -k_{-I}I + k'_I S + D\nabla^2 I,$$

$$\frac{\partial R^*}{\partial t} = -k_{-2}IR^* + k_2AR,$$

$$\frac{\partial R}{\partial t} = -k_{-1}IR + k_{1a}A + k_{-2}IR^* - k_2AR. \qquad (S1)$$

The rate constants are denoted as $k_{-A},\ k_A, k_{-I}, k'_I, k_{-2}, k_2, k_{-1}, k_{1a}, k_{-2}$ and k_2. The diffusion coefficient of the inhibitor I in the cytosolic region is represented by D. Note that, by the first and the second terms in the RHS of the fourth equation, the activator A and the inactivator I produce and degrade the inactive state of the response element R, respectively, which contribute to the amplification of the output signal R^* [5,6]. The capability of the system to show spatial FCD in a static spatially-graded stimulus [5,6] can be understood by the spatial FCD symmetry of the model; at a steady state, i.e, $\frac{\partial A}{\partial t} = \frac{\partial I}{\partial t} = \frac{\partial R^*}{\partial t} = \frac{\partial R}{\partial t} = 0$, solutions of Eqs. S1 are invariant under a scale transformation

$$(A, I, R^*, R, S)\ \rightarrow (pA, pI, R^*, R, pS)\ \ (p > 0). \qquad (S2)$$

However, without assuming a steady state, the scale invariance does not hold, and thus the system does not show the temporal FCD property. Another model proposed by the authors combines the similar circuit to Eqs. S1 with an amplification module consisting of new variables, T and T_{in} (Fig. 3B in [6]):

$$\frac{\partial A}{\partial t} = -k_{-A}A + k_AS,$$

$$\frac{\partial I}{\partial t} = -k_{-I}I + k'_IS + D\nabla^2 I,$$

$$\frac{\partial R^*}{\partial t} = -k_{-R}IR^* + k_RA(R_{tot} - R^*),$$

$$\frac{\partial T}{\partial t} = k_T\frac{R^*T_{in}}{K_T + T_{in}} - k_\rho T,$$

$$\frac{\partial T_{in}}{\partial t} = -k_T\frac{R^*T_{in}}{K_T + T_{in}} + k_\rho T + \sigma + k_\sigma T - \gamma_\sigma T_{in}, \qquad (S3)$$

where $k_{-A},\ k_A, k_{-I}, k'_I, k_{-R}, k_R, k_T, k_\rho, k_\sigma, K_T$ and γ_σ are rate constants. D and σ are the diffusion coefficient and the rate of constant supply of T_{in}, respectively. The total amount of the response elements (R_{tot}) is conserved. In addition to a feedforward circuit described by the first three equations, the fourth and fifth equations compose an amplification module based on a positive feedback loop, which locates at the downstream of the feedforward circuit [6]. The system (Eqs. S3) shows essentially the same behavior as the previous one (Eqs. S2) from the point of view of the response rescaling. Namely, the equations are scale invariant upon a scale transformation,

$$(A, I, R^*, T, T_{in}, S)\ \rightarrow (pA, pI, R^*, T, T_{in}, pS)\ \ (p > 0) \qquad (S4),$$

only when the system is in a steady state. Thus, the temporal FCD is not achieved. However, the system shows the temporal FCD if we assume the kinetics of the third equation is sufficiently fast. In this case, the third equation becomes an algebraic equation as

$$R^* = \frac{k_A A}{k_A A + k_{-R} I} R_{tot}. \quad \text{(S5)}$$

Then, the system is now invariant to the scale transformation (Eqs. S4) without assuming a steady state and therefore shows both spatial and temporal FCDs.

Following this scheme, Wang et al. [7] have proposed a model in which the upstream module is the same as the first three equations in Eqs. S3 while the downstream amplification module is replaced by another one. Hence, the same logic as in Eqs. S3 applies to their model. Namely, it satisfies the spatial FCD symmetry without modification and, by assuming fast kinetics of one variable, it can achieve both symmetries.

(ii) A balanced inactivation model [8]

Levine et al. have proposed a LEGI-based model which, instead of assuming downstream amplification module as in Eqs. S3, introduces a hypothetical mutual inhibition between chemical components to describe amplification and adaptation [8]. The governing equations are

$$\frac{\partial A}{\partial t} = k_a S - k_{-a} A - k_i A B_m \text{ at the membrane,}$$

$$\frac{\partial B_m}{\partial t} = k_b B - k_{-b} B_m - k_i A B_m \text{ at the membrane, and}$$

$$\frac{\partial B}{\partial t} = D \nabla^2 B \text{ in the cytosol} \quad \text{(S6),}$$

with a boundary condition

$$D \frac{\partial B}{\partial n} = k_a S - k_b B,$$

where the derivative is the ourward pointing normal derivative of the cytosolic component *B*. The rate constants are represented by $k_a,\ k_{-a}, k_i, k_b$ and k_{-b}, respectively. *D* is the diffusion coefficient of the cytosolic component *B*. Due to the mutual inhibition term ($k_i A B_m$) in the first and second equations, the system does not show any scale invariance upon scale change in the input stimulus, i.e., $S \to pS$. As a result, the system shows neither temporal FCD (Fig. 1 in [8]) nor spatial FCD. In fact, the output level *A* in a steady state does not show perfect adaptation and depends on the absolute level of the input stimulus *S* as shown analytically in [8].

(iii) Ultrasensitive LEGI model [5]

An alternative LEGI-based model has been proposed by Nakajima et al [5] to describe the responses in traveling wave stimuli. The governing equations are

$$\frac{\partial A}{\partial t} = k_a S - \gamma_a A,$$

$$\frac{\partial I}{\partial t} = k_i S - \gamma_i I + D \nabla^2 I,$$

$$\frac{\partial R}{\partial t} = Ak_A \frac{R_{tot} - R}{K_A + (R_{tot} - R)} - Ik_I \frac{R}{K_I + R} \quad \text{(S7)},$$

where $k_a,\ \gamma_a, k_i,\ \gamma_i, k_A, K_A, k_I$ and K_I are rate constants. D is the diffusion coefficient of the inhibitor I. This model has essentially the same upstream module as the model (S3), but the output signal R is now regulated by the functions of Michaelis-Menten form, which is critical for the highly asymmetric response to spatially-symmetric traveling wave stimuli [5]. In the same way as the previous model (Eqs. S3), this model at a steady state satisfies the spatial FCD symmetry under the transformation,

$$(A, I, R, S) \rightarrow (pA, pI, R, pS)\ \ (p > 0) \quad \text{(S8)}.$$

This explains the rescaled response to a gradient stimulus of the model [5]. Also in the same way as the previous model (Eqs. S3), by assuming fast kinetics of the third equation, the equations satisfie the temporal FCD symmetry.

(iv) LEGI-BEN model [9]

Tang et al. [9] have recently proposed a model based on the so-called LEGI-BEN scheme where a LEGI module is combined with a downstream excitable system [10]. Although the primary interest of the line of research is to connect gradient sensing with migration, here we focus only on the upstream LEGI module and its rescaling property. The model equations are

$$\frac{\partial A}{\partial t} = k_a S - \gamma_a A,$$

$$\frac{\partial I}{\partial t} = k_i S - \gamma_i I + D\nabla^2 I,$$

$$\frac{\partial R}{\partial t} = k_R \frac{k_A + A}{k_I + I} - \gamma_R R \quad \text{(S9)},$$

where $k_a, \gamma_a, k_i, \gamma_i, k_R, k_A, k_I$ and D are constant parameters. The model shows neither the spatial nor temporal FCD symmetry on its own. However, the model can show spatio-temporal FCD under a condition without assuming fast kinetics of any variable like we have done in the previous sections. A characteristic feature of the model is that the inhibitor I down-regulates the output R by suppressing the synthesis of R by the activator A, which is described by the first term on the right hand side of the third equation. Because of the term, in the limit of $k_A, k_I \rightarrow 0$ (or the limit of large input), the model becomes to satisfy both space and temporal FCD symmetry under the transformation,

$$(A, I, R, S) \rightarrow (pA, pI, R, pS)\ \ (p > 0) \quad \text{(S10)}.$$

In the original LEGI-BEN model, the rescaled output from the upstream module is fed into the downstream system (i.e., the excitable system) and therefore assures rescaled behaviors of the entire system.

A possible molecular mechanism for the nonlinear activation function

In the scale-invariant LEGI model (Eqs. 1 in the main text), we adopted a nonlinear function $S^n/(S^n + (KB)^n)$ in which the inhibitor *B* suppresses the activator *A* by lowering the sensitivity to the input *S*, not by degrading the activator *A* directly. Below, we show how the sensitivity control can emerge in a biochemical network. Let us introduce a path inhibition model as follows:

$$\frac{\partial M}{\partial t} = k_m S - k_{-m} B M$$
$$\frac{\partial A}{\partial t} = k_a \frac{M^n}{M^n + K^n} - k_{-a} A,$$
$$\frac{\partial B}{\partial t} = k_b S - k_{-b} B + D(\langle B \rangle - B) \quad \text{(S11)},$$

where k_m and k_{-m} represent the rate constants of the equation for *M* and the other parameters and variables are the same as in Eqs. 1. In this model, a signaling component *M* mediates excitatory regulation from the input *S* to the activator *A*, and the inhibitor *B* suppresses the mediator *M* instead of the activator signal directly (hence it is called a 'path inhibition' model). When the kinetics of *M* is fast, the mediator level *M* is approximated by *S*/*B* (Here we assume $k_m/k_{-m} = 1$ for simplicity). By substituting *M* = *S*/*B* into the equation for *A*, we obtain the nonlinear sensitivity control function. The resultant model is equivalent to the scale-invariant LEGI model (Eqs. 1).

References

1. Parent CA, Devreotes PN. A Cell ' s Sense of Direction. Science (80-). 1999;284: 765–770.
2. Goentoro L, Shoval O, Kirschner MW, Alon U. The Incoherent Feedforward Loop Can Provide Fold-Change Detection in Gene Regulation. Mol Cell. Elsevier Ltd; 2009;36: 894–899. doi:10.1016/j.molcel.2009.11.018
3. Shoval O, Goentoro L, Hart Y, Mayo A, Sontag E, Alon U. Fold-change detection and scalar symmetry of sensory input fields. Proc Natl Acad Sci U S A. 2010;107: 15995–16000. doi:10.1073/pnas.1002352107
4. Adler M, Mayo A, Alon U. Logarithmic and Power Law Input-Output Relations in Sensory Systems with Fold-Change Detection. PLoS Comput Biol. 2014;10: e1003781. doi:10.1371/journal.pcbi.1003781
5. Nakajima A, Ishihara S, Imoto D, Sawai S. Rectified directional sensing in long-range cell migration. Nat Commun. Nature Publishing Group; 2014;5: 1–14. doi:10.1038/ncomms6367
6. Levchenko A, Iglesias PA. Models of eukaryotic gradient sensing: application to chemotaxis of amoebae and neutrophils. Biophys J. Elsevier; 2002;82: 50–63. doi:10.1016/S0006-3495(02)75373-3
7. Wang CJ, Bergmann A, Lin B, Kim K, Levchenko A. Diverse sensitivity thresholds in dynamic signaling responses by social amoebae. Sci Signal. 2012;5: ra17. doi:10.1126/scisignal.2002449
8. Levine H, Kessler DA, Rappel W-J. Directional sensing in eukaryotic chemotaxis: a balanced inactivation model. Proc Natl Acad Sci U S A. 2006;103: 9761–9766. doi:10.1073/pnas.0601302103
9. Ming Tang#1, Mingjie Wang1, 3, Changji Shi2, Pablo A. Iglesias1, 2, Peter N. Devreotes1, 4 A, Huang C-H. Evolutionarily Conserved Coupling of Adaptive and Excitable Networks Mediates Eukaryotic Chemotaxis. Nat Commun. 2015;29: 997–1003. doi:10.1016/j.biotechadv.2011.08.021.Secreted
10. Iglesias P a., Devreotes PN. Biased excitable networks: How cells direct motion in response to

gradients. Curr Opin Cell Biol. Elsevier Ltd; 2012;24: 245–253. doi:10.1016/j.ceb.2011.11.009
